# Integrated genomic and prospective clinical studies show the importance of modular pleiotropy for disease susceptibility, diagnosis and treatment

**DOI:** 10.1186/gm534

**Published:** 2014-02-26

**Authors:** Mika Gustafsson, Måns Edström, Danuta Gawel, Colm E Nestor, Hui Wang, Huan Zhang, Fredrik Barrenäs, James Tojo, Ingrid Kockum, Tomas Olsson, Jordi Serra-Musach, Núria Bonifaci, Miguel Angel Pujana, Jan Ernerudh, Mikael Benson

**Affiliations:** 1 The Centre for Individualised Medicine, Department of Clinical and Experimental Medicine, Linköping University, 58185 Linköping, Sweden; 2 Clinical and Experimental Medicine, Faculty of Health Sciences, Division of Clinical Immunology, Unit of Autoimmunity and Immune Regulation, Linköping University, 58185 Linköping, Sweden; 3 Department of Clinical Neurosciences, Karolinska Institutet and Centrum for Molecular Medicine, 17177 Stockholm, Sweden; 4 Cancer and Systems Biology Unit, Catalan Institute of Oncology, IDIBELL, L’Hospitalet del Llobregat, 08908 Barcelona, Spain

## Abstract

**Background:**

Translational research typically aims to identify and functionally validate individual, disease-specific genes. However, reaching this aim is complicated by the involvement of thousands of genes in common diseases, and that many of those genes are pleiotropic, that is, shared by several diseases.

**Methods:**

We integrated genomic meta-analyses with prospective clinical studies to systematically investigate the pathogenic, diagnostic and therapeutic roles of pleiotropic genes. In a novel approach, we first used pathway analysis of all published genome-wide association studies (GWAS) to find a cell type common to many diseases.

**Results:**

The analysis showed over-representation of the T helper cell differentiation pathway, which is expressed in T cells. This led us to focus on expression profiling of CD4^+^ T cells from highly diverse inflammatory and malignant diseases. We found that pleiotropic genes were highly interconnected and formed a pleiotropic module, which was enriched for inflammatory, metabolic and proliferative pathways. The general relevance of this module was supported by highly significant enrichment of genetic variants identified by all GWAS and cancer studies, as well as known diagnostic and therapeutic targets. Prospective clinical studies of multiple sclerosis and allergy showed the importance of both pleiotropic and disease specific modules for clinical stratification.

**Conclusions:**

In summary, this translational genomics study identified a pleiotropic module, which has key pathogenic, diagnostic and therapeutic roles.

## Background

Medical research typically focuses on individual diseases and individual genes. This focus is complicated by many patients having more than one disease, the heterogeneity of diseases as well as the involvement of thousands of genes, many of which are shared by more than one disease. Several observations point to the need to systematically investigate the pathogenic roles of shared, or pleiotropic genes: their importance is well established in model organisms and monogenic diseases [1-3]. For example, mutations in the same gene, *ARX*, cause two highly diverse diseases, namely ambiguous genitalia and lissencephaly, a disease of the brain (Online Mendelian Inheritance in Man no. 300215). Genome-wide association studies (GWAS) have also shown the importance of pleiotropy in complex diseases [4-8]. Identification of pleiotropic genes by GWAS of complex diseases is limited by the relatively modest effects of genetic variants [5] and because epigenetic causes also play important roles [9,10]. Since mRNA expression can be affected by both genetic and epigenetic variants, expression profiling may be an optimal way to identify pleiotropic genes. In order to get an overview of the large number of genes involved in complex diseases, a module-based strategy can be applied. Briefly, this strategy is based on the concept that disease-associated genes are not randomly distributed when mapped on the human protein-protein interaction (PPI) network. Instead, they tend to co-localize and form modules in the PPI network [11-22]. The advantages of modules are that they are thought to contain a limited number of the most disease-relevant genes, and that a functional overview of disease mechanisms can be obtained by pathway analysis.

In this study, we used a module-based approach to systematically investigate the pathogenic, diagnostic and therapeutic roles of pleiotropic genes. The approach was applied to public expression profiling data from different diseases. Because gene expression varies in different cell types, such data should ideally be derived from the same cell type. To reach this ideal, we first performed pathway analysis of all published GWAS to find a cell type common to many diseases. This resulted in the identification of the T helper (Th) cell differentiation pathway, which is expressed in T cells. We therefore focused on expression profiling of CD4^+^ T cells from highly diverse inflammatory and malignant diseases. Analysis of the expression profiling data, which were independent from the GWAS data, resulted in the identification of pleiotropic genes. These genes were highly interconnected and formed a pleiotropic module. Despite being derived from the same cell type, this module was associated with a general increase in disease susceptibility: it was enriched for highly diverse pathways, as well as genetic variants described by all GWAS and cancer studies. The module was also enriched for known diagnostic and therapeutic targets. Moreover, the pleiotropic or disease-specific modules could be used to stratify patients in prospective clinical studies of multiple sclerosis and allergy. Our results show the clinical importance of pleiotropic genes as well as of translational genomics.

## Materials and methods

### Ethics statement

The studies were approved by the ethics board of University of Gothenburg and the ethics board of University of Linköping. All participants provided written consent for participation.

### Study subjects

The study comprised two different sets of experimental data: 1) response to cortisone treatment in 48 seasonal allergic rhinitis (SAR) patients, of which 8 were classified as high responders (HRs; see below for definitions) and 8 as low responders (LRs; 5 men and 11 women, mean age 35.6 ± 2.3 years); and 2) response to natalizumab treatment in 50 multiple sclerosis (MS) patients, of which 8 were classified as HRs and 8 as LRs (14 men and 2 women, mean age 35.6 ± 1.5 years). The SAR patients and healthy donors were of Swedish origin and recruited at The Queen Silvia Children’s Hospital, Gothenburg. SAR was defined by a positive seasonal history and a positive skin prick test or by a positive ImmunoCap Rapid (Phadia) to birch and/or grass pollen. Patients with perennial symptoms or asthma were not included. The healthy subjects did not have any history for SAR and had negative ImmunoCap Rapid tests. The MS patients suffering from relapsing-remitting disease were of Swedish origin and recruited at the Department of Neurology at Linköping University Hospital. Patients had definite MS according to the McDonald criteria. All the microarray data from this study have been deposited under the Gene Expression Omnibus superseries GSE44966.

### Definition of low and high responders to treatment

An initial cohort of 48 allergic patients was asked to mark their symptoms (rhinorrhea, congestion, and itching) on a visual analogue scale (VAS) before and after two weeks of nasal treatment with fluticasone. We defined LRs/HRs from the decrease in symptom scores when treated for two weeks in the pollen season, using previously described methods [23]. Fifty patients were included in the MS group and treated with natalizumab (NZB; Tysabri®); patients were classified as LRs (n = 8) if they had experienced at least one relapse during this follow-up period and as HRs if this had not occurred (n = 42, of which 8 were selected because of age, gender and disease matching to the LR group). All patients were followed for three years, except two LRs, who were followed for two and one year, respectively. The annual relapse rate was 0.65 ± 0.26 for LRs and 0 for HRs. Samples analyzed for gene expression were taken prior to clinical natalizumab treatment. Both groups had comparable age distribution (mean age 37.3 ± 5.8 (standard deviation) years for LRs and 33.9 ± 6.2 for HRs), Expanded Disability Status Scale score (LR, median 2.5, range 0.0 to 7.0; HRs, 2.0, range 1.0 to 5.0), disease duration (LR, mean 9.1 ± 6.6 (standard deviation) years; HR, 9.9 ± 6.3 years) and gender distribution (seven males in both groups) at the time of inclusion.

### Bioinformatics analyses

Public data were downloaded from the Gene Expression Omnibus that met the following criteria on 31 December 2012 (Table S6 in Additional file 1): i) expression profiling of CD4^+^ T cells from healthy controls and patients with T-cell-related non-virus diseases; ii) at least five samples per disease and controls; and iii) the patients were not drug treated. In this study, data were quantile-normalized and log-transformed. The GCs and natalizumab materials were corrected for potential batch effects using COMBAT [24], with phenotype and stimulation as covariates. We mapped all probes to the corresponding genes and in cases where multiple probes for the same gene were present we used the median probe levels. Differential expression was computed using the LIMMA package in R. Classification by the LASSO was performed by the MatLab function lassoglm in Statistics Toolbox choosing λ from the minimum deviance of leave-one-out cross-validation starting from all the measured genes of the platform. To determine if a set of genes was differentially expressed we used the mean of the squares of the student *t*-test statistic. This particular choice was made since its theoretical distribution is *X*^2^(*n* - 1) and thereby the size effect was easy to interpret [25]. However, for calculating the *P*-values we still used permutation test of the mean value of the squared student *t*-values. We also performed complementary analysis using the log *P*-values with similar results. Permutation tests were performed using 10^6^ permutations if nothing else is stated, and for the estimation of small *P*-values we approximated the distribution using [26]. The enrichments of GWAS genes, cancer genes, mouse knockout phenotypes, therapeutic targets, and biomarkers was also controlled for connectivity biases. This was performed by randomly sampling 158 genes from the STRING network with the same median (182) and minimal degree (39) as the pleiotropic module and repeating this procedure 10^6^ times. The false discovery rate (FDR) was determined using the Benjamini Hochberg [27] correction method. The size effect was represented by fold enrichments (FEs), which is defined here for a specific feature and test set by the frequency of genes in the tested set with a certain feature divided by the frequency of the feature among all annotated genes. For example, we found the GWAS frequency 33.5% (53/158) among the pleiotropic genes, whereas this in the background of all genes is about 10.2% (2,298/22,500), and therefore FE = 33.5/10.2 = 3.3.

### Databases

In order to test the robustness of the pleiotropic module we downloaded the latest versions (15 November 2013) of five different databases covering different aspects of the human interactome, namely HPRD [28], Reactome [29], Intact [30], HI2 (updated union of HI-2011, HI-2009 and HI-2005 from [31]) [30], and a high confidence database [32]. For each of these databases we then first computed the shortest distances between all pairs of proteins in the pleiotropic module that were also present in the largest connected component. Then we randomly selected the same number of genes from the largest connected component and similarly computed their shortest paths. We repeated the random selection procedure 1,000 times and calculated *P*-values using a Wilcoxon test of the mean values of the shortest paths. The enrichment of pathways within GWAS genes was tested using all pathways (as of 15 November 2013) in three different annotation databases, namely Kyoto Encyclopedia of Genes and Genomes (KEGG) [33], Ingenuity Pathway Analysis (IPA; Ingenuity® Systems, [34]), and Gene Ontology (GO) [35]. These enrichment analyses were performed using all human genes in NCBI (National Center for Biotechnology Information) as background. The analysis of mouse knockout phenotypes was performed by downloading phenotypic information from [36] as of 31 January 2013. The analysis of therapeutic targets and biomarkers was also performed by first downloading all therapeutic target drugs and all genes annotated as markers for disease or prognosis in the IPA database. *P*-values for enrichment were calculated using Fisher's exact test (FET) using all genes as background (n = 22,500 for human and n = 6,964 for mice).

For the correlation analysis between the basal expression of the 158 genes and the drug responses across cancer cell lines, data were downloaded from the Genomics of Drug Sensitivity project [37]. This dataset included IC50 values for a total of 131 drugs that were assessed in a panel of 638 human cancer cell lines. In combination with this information, gene expression data from the same study (corresponding to the basal transcriptional profiles for 595 cell lines and publicly available on the ArrayExpress reference E-MTAB-783) were analyzed. Normalized expression values were obtained using the RMA algorithm [38]. Non-annotated probes were removed from the analysis and correlations were computed using the Pearson’s correlation coefficient (PCC). For the null distribution, 1,000 sets of 158 genes randomly selected from the same microarray dataset were analyzed.

### Disease-associated network modules

The network and modules were constructed using a modified version of a previously described method [39]. First, all maximal cliques [29] were extracted from the human PPI database STRING [40] (version using 9.03 interactions with a confidence score ≥0.7). Then, each clique was assigned a weight, defined as the sum of the -log(*P*-values) for all the genes in the clique, based on the differential expression analysis (henceforth referred to as the real weight). To assess how the weight for each clique differed from the null distribution, the *P*-values for all the genes in the differential expression analysis were randomized, and the weight for each clique was again determined by the same method. This process was repeated 10,000 times, to give a null distribution of weights for each clique. The significance of each clique’s weight was defined as the fraction of random permutations that resulted in a higher weight than the real weight. Disease modules were then constructed by mapping the cliques that had a significantly higher weight than expected by the null distribution (*P* < 0.01). This analysis resulted in a module associated to each disease. We then identified the intersection between the disease modules. To determine how the size of the intersection differed from random, we repeated the above analysis 100 times using randomized *P*-values. This resulted in 100 disease modules for every disease. We then determined the intersection between these 100 sets of disease modules and compared this to the real intersection. Furthermore, we used these 100 sets of disease modules to identify genes that were represented in more disease modules than expected by chance. Like before, the *P*-value of each gene was equivalent to the ratio of random permutations that included the gene in as many or more disease modules as the disease modules that resulted from real (non-randomized) data. The R code for the identification of disease modules from interactome and *P*-values can be found in Additional file 2, and the output disease modules are provided in Table S22 in Additional file 1.

### Culturing of CD4^+^ T cells from patients with SAR

Challenges with allergen or allergen plus glucocorticoids (GCs) were performed as previously described [41-43]. Briefly, peripheral blood mononuclear cells were prepared from fresh blood by means of centrifugation in Ficoll-Hypaque, washed, and stimulated with allergen extract (100 μg/ml; ALK-Abelló A/S Hørsholm, Denmark), or allergen plus hydrocortisone (10^-7^ M; Sigma-Aldrich, St. Louis, Missouri, USA). After 17 hours and 7 days, respectively, of incubation at 37°C and 5% CO_2_, the supernatant was removed and CD4+ T cells enriched. The RNA samples were then analyzed using Agilent Sureprint G3 Human Gene Expression.

### Culturing of CD4^+^ T cells from patients with MS

Briefly, mononuclear cells were thawed from liquid N2. Next, CD4^+^ T cells were separated using the Isolation Kit II (Miltenyi Biotech, Bergisch Gladbach, Germany). Purity of samples was typically >97%. The CD4^+^ T cells were split into three cultures and an unstimulated culture served as baseline control. Two cultures were stimulated with pre-coated anti-CD3/-CD28 monoclonal antibodies (0.1 μg/ml). Cells were cultured for 48 hours in media consisting of Iscove's modified Dulbecco's medium (IMDM) supplemented with 5% fetal calf serum (Sigma-Aldrich), L-glutamine (292 mg/ml; Sigma-Aldrich), sodium bicarbonate (3.024 mg/ml; Sigma-Aldrich), penicillin (50 IE/ml; Cambrex, East rutherford, New Jersey, USA), streptomycin (50 μg/ml; Cambrex), and 100× non-essential amino acids (Gibco BRL, New York, USA). One of these cultures was supplemented with Tysabri® at a final concentration of 25 μg/ml. After culturing, cells were lysed using TRI Reagent (MRC, London UK). Total RNA was extracted according to the manufacturer’s instructions. RNA quality and quantity were assessed with the NanoDrop ND-1000 (NanoDrop Technologies Wilmington, New Jersey, USA). The Agilent Sureprint G3 Human Gene Expression 8x60k was used for gene expression analysis (Agilent Technologies, Santa Clara, California, USA).

## Results and discussion

### Pathway analysis of GWAS leads to the selection of T cells to study the expression of pleiotropic genes

In order to identify pleiotropic genes at the transcriptomics level, the expression of those genes should ideally be measured in the same cell type. Because expression varies in different cell types, we performed pathway analysis of GWAS to find a pleiotropic pathway. The aim was to use that pathway to find a pleiotropic cell type that was accessible for independent expression profiling studies. This led to the identification of Th differentiation, which is expressed in T cells, as described below.

**Figure 1:**
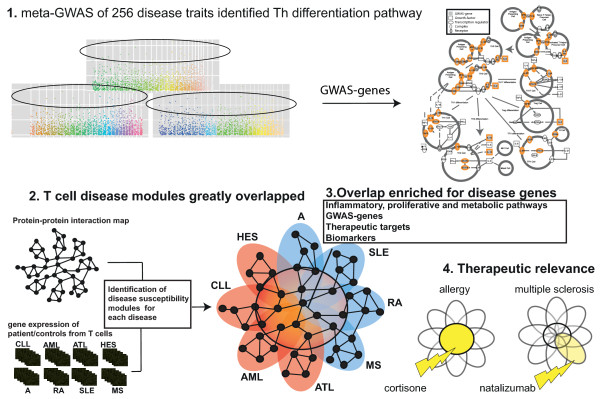
**Overview of the workflow used in this study.** 1) Meta-GWAS of 256 diseases and disease traits (left) revealed Th differentiation (right) to be the pathway most enriched for GWAS genes. 2) Identification of disease modules in eight CD4^+^ T-cell-associated diseases (A, allergy; AML, acute myelogenous leukemia; ATL, adult T cell leukemia; CLL, chronic lymphocytic leukemia; HES, hypereosinophilic syndrome; MS, multiple sclerosis; RA, rheumatoid arthritis; SLE, systemic lupus erythematosus). These modules partially overlapped and formed a pleiotropic module. The pleiotropic module is marked using a solid black circle. 3) The pleiotropic module was highly enriched for genes relevant to many diseases. 4) Prospective clinical studies of multiple sclerosis and seasonal allergic rhinitis showed that pleiotropic and disease-specific genes could stratify patients for individualized medication.

First, meta-analysis of GWAS combined with pathway enrichment analysis was performed (Figure 1, analysis 1). We downloaded GWAS data compiled by the National Human Genome Research Institute [44]: this included 256 diseases and traits, and 2,298 gene loci harboring potentially associated SNPs (mapping inter-genic SNPs to the nearest upstream and downstream genes at a significance threshold *P* < 10^-5^; see Table S1 in Additional file 1 and Figure S2 in Additional file 3 for disease/trait distribution). These genes, which represent potential associations with diseases or disease traits, are henceforth referred to as 'GWAS genes' (Table S2 in Additional file 1). Next, using a large curated database (Ingenuity® Systems [34]), the Th cell differentiation pathway (Figure S2 in Additional file 3) was identified as the most enriched in the GWAS genes measured by the *P*-values from a FET (n = 38, FE = 5.3, Bonferroni-corrected *P* < 10^-15^; Table S3 in Additional file 1 shows all genes and pathways). Th differentiation results in different T-cell subsets, such as Th1, Th2 and Th17, which are thought to have key roles in immune-related diseases, but are also associated with many other diseases [45-48].

In order to test the importance of Th differentiation in diseases that were not *a priori* immune related, and to minimize knowledge biases related to immune diseases, we performed six complementary analyses. These analyses supported the importance of Th differentiation.

First, we queried the GO [35] database for enrichments of the GO process T-cell differentiation and found that this was the case (*P* < 2 × 10^-6^, FET). Second, we tested that the corresponding pathway (T-cell receptor pathway, *P* < 4 × 10^-3^) was enriched for the GWAS genes using KEGG (Table S4 in Additional file 1; see Figure S3 in Additional file 3 for a comparison of the overlap of IPA, KEGG, and GO). Third, we manually classified the disease traits into immune and non-immune (Tables S1 and S2 in Additional file 1) and again found the Th differentiation to be highly enriched also in the non-immune disease category (*P* < 1 × 10^-7^; Table S5 in Additional file 1). Fourth, we repeated the analyses for all genes within each block, where the corresponding gene is in linkage disequilibrium [49] (Th differentiation, *P* < 3 × 10^-7^; Tables S6 and S7 in Additional file 1). Fifth, we repeated the analysis for 446 genes with known somatic cancer mutations [50], henceforth referred to as 'cancer genes' (Th differentiation, *P* < 5 × 10^-4^; Tables S8 and S9 in Additional file 1). Sixth, 4,613 genes annotated to disease from the Online Mendelian Inheritance in Man database [51] (visited on 12 November 2013; Th differentiation, *P* < 2 × 10^-11^; Tables S10 and S11 in Additional file 1).

Since the Th differentiation pathway is expressed in CD4^+^ T cells, we proceeded to analyze expression profiling data from this cell type. Note that the analyses below are based on all genes, and not only genes associated with Th differentiation.

### A pleiotropic module in CD4^+^ T-cell-associated diseases was associated with generally increased disease susceptibility

In order to identify pleiotropic genes, publicly available gene expression microarray data for eight diseases were analyzed: four inflammatory diseases (allergy, multiple sclerosis, rheumatoid arthritis, and systemic lupus erythematosus), and four malignant or proliferative diseases (acute myelogenous leukemia, adult T cell leukemia, chronic lymphocytic leukemia, and hypereosinophilic syndrome; Figure 1, analysis step 2; Table S12 in Additional file 1; the diseases were chosen because they met the criteria described in Materials and methods).

Despite the diversity of these diseases, we found that the genes that were differentially expressed between patients and healthy controls were not generally dispersed when mapped on the human PPI network. Instead, the genes from each disease formed modules, which partially overlapped. The overlapping genes were highly interconnected and formed a pleiotropic module. This module was associated with a wide variety of diseases, as shown by pathway analysis, phenotypes resulting from mouse knockout studies, as well as an enrichment of GWAS and cancer genes. The analyses are detailed below.

We first confirmed that patients and controls could be correctly classified using the LASSO approach (10^-16^ < *P* < 10^-3^, permutation test; Figure S4 in Additional file 3; Table S12 in Additional file 1; see Materials and methods for details).

Differentially expressed genes for each of the eight diseases were identified and subsequently mapped on a network of known and predicted human PPIs from the STRING database [40] (Materials and methods). For each disease, we identified subnetworks of highly inter-connected and differentially expressed genes, using our previously published method [39]. Such interconnected and differentially expressed genes are henceforth referred to as disease modules. In our study, this led to the identification of eight different sets of partially overlapping and interconnected disease modules, which each consisted of 1,215 to 1,933 genes.

**Figure 2:**
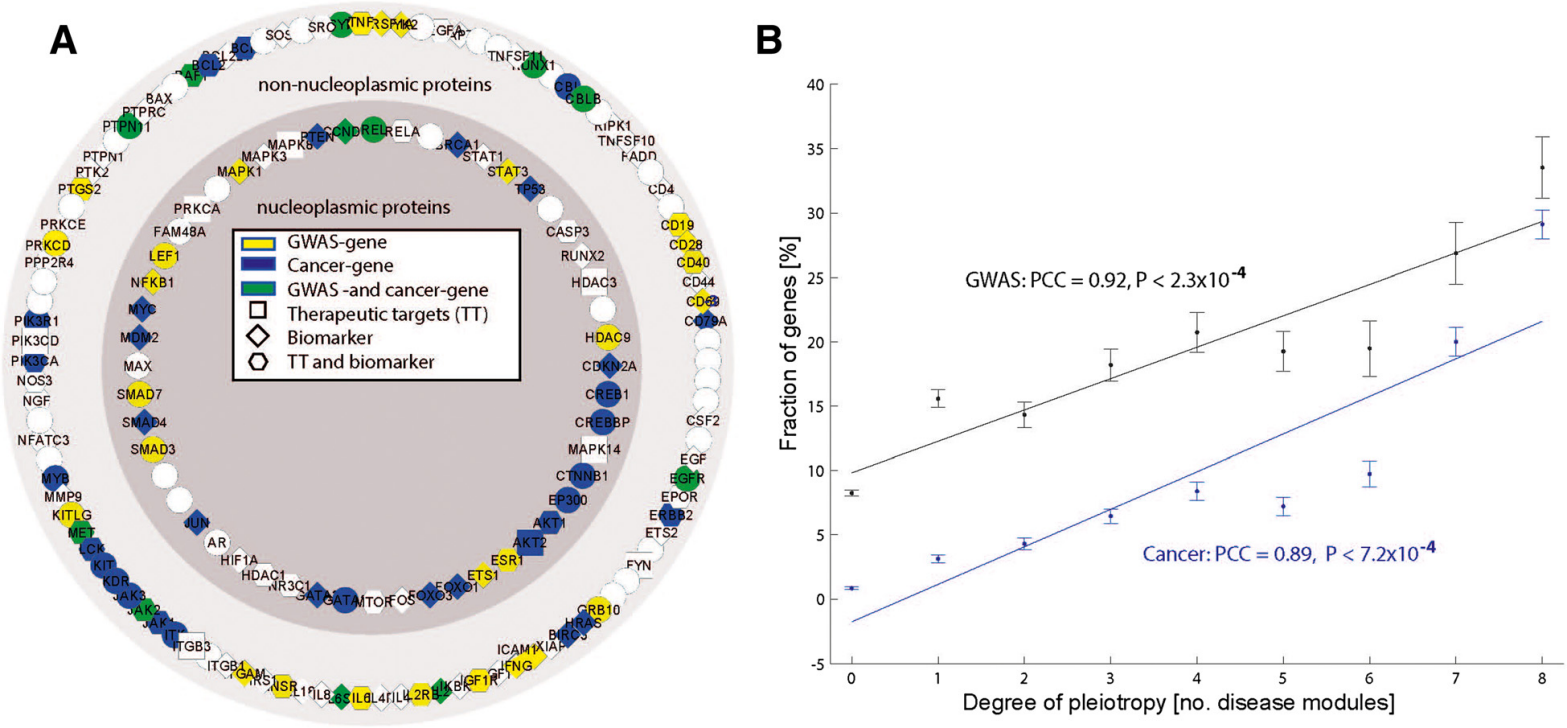
**The pleiotropic module was enriched for GWAS genes, cancer genes, as well as therapeutic targets and biomarkers. (A)** Schematic representation of the pleiotropic module. The inner circle represents nucleoplasmic genes and the outer non-nucleoplasmic genes according to Gene Ontology. Therapeutic targets are marked with squares, biomarkers with tilted squares, and hexagons represent both. Node colors code for GWAS genes (yellow), cancer genes (blue), both (green). For clarity we do not show the 7,144 interactions between the pleiotropic module genes. **(B)** Strongly positive correlations were found between the fraction of GWAS genes and cancer genes in the modules versus the number of disease modules. PCC, Pearson's correlation coefficient.

We examined if the disease modules for each of the eight different diseases overlapped significantly (Materials and methods). We found a highly significant overlap of 158 genes (Table S13 in Additional file 1) in all eight disease modules (FE = 7.0, *P* < 10^-61^ permutation test; Materials and methods; Figure 2). The 158 genes were highly interconnected and had 7,144 interactions between each other. By contrast, the mean number of connections between the 158 genes in the disease module and 158 random genes was only 376 (*P* < 10^-300^, permutation test). As different PPI databases have different inclusion criteria, we tested if the overlapping 158 genes had a lower mean shortest path among each other than 158 random genes, using five different databases covering different aspects of the human interactome (Materials and methods). This analysis showed that, for all five analyzed data sets, we had significantly lower mean shortest paths among the 158 genes in the overlap of all the disease modules than expected (*P* < 10^-3^ in four databases and *P* < 0.011 for one database using one-sided Wilcoxon rank test; see Materials and methods and Figure S5 in Additional file 3 for details).

Henceforth, the 158 genes are referred to as the 'pleiotropic module'. Despite the expression profiling data being derived from T cells, pathway analysis of the pleiotropic module showed enrichment not only of inflammatory pathways, but also of multiple other disease-associated pathways (Table S14 in Additional file 1 shows results for all pathways enriched in the pleiotropic module). Because of the pleiotropy and interconnectivity of the pathways, we hypothesized that the genes in the pleiotropic module would generally increase disease susceptibility. This was supported by analysis of the mouse knockout database, which showed that a wide range of phenotypes and diseases resulted from knock out of genes in the pleiotropic module (Bonferroni corrected *P*-values from *P* < 10^-50^, FET; Table S15 in Additional file 1). The pleiotropic module was also significantly enriched for both GWAS and cancer genes (GWAS, n = 53, FE = 3.3, *P* < 10^-15^; cancer, n = 47, FE = 15.4, *P* < 10^-41^; Figure 1). Moreover, we tested the general disease importance of the pleiotropic module by only studying GWAS for the 158 diseases and disease traits that were not *a priori* associated with immune-related diseases and cancer (see Table S1 in Additional file 1 for disease categorizations), which then consisted of 1,437 genes. e found 19 of the pleiotropic genes were significantly associated with these diseases and disease traits (FE = 1.9, *P* < 4 × 10^-3^).

We also confirmed that the enrichment was significantly more than expected by the degree of the corresponding genes (Materials and methods; Table S16 in Additional file 1). Then, in order to test if the enrichment was dependent on the definition of the pleiotropic module, we performed a correlation analysis: we correlated the fraction of disease susceptibility genes and the number of disease modules for each gene and found a strong significant positive PCC for both GWAS (PCC = 0.91, *P* < 4 × 10^-4^) and cancer (PCC = 0.94, *P* < 2 × 10^-4^) genes (Figure 1B). Thus, the enrichment was not due to the definition of the pleiotropic module.

A limitation in the above analyses of SNPs from public databases was that those SNPs had been defined based on genome-wide significance levels, although we had only examined 158 genes. In order to increase statistical power, we also analyzed the 158 genes for nominally significant associations in the original GWAS data from MS [52], comprising approximately 25,000 individuals. We defined a gene to be nominally significant if it harbored at least one SNP with a nominally low *P*-value. We found that the pleiotropic genes were enriched for MS SNPs using *P*-values in the range 10^-5^ to 0.05 (Figure S6 in Additional file 3). For example, we found that 60% (89/148) of the assayed probes had at least one SNP with *P*-value <0.05 (*P* < 2 × 10^-6^, FET) and 40% had SNPs with a *P*-value <0.01 (*P* < 10^-12^). Furthermore, by examining all assayed SNPs (n = 4,990) of the pleiotropic genes we found that 150 SNPs for 51 unique pleiotropic genes (Table S17 in Additional file 1) were disease associated using a 20% FDR, which represents a huge increase from the five genes originally reported using genome-wide significance criterion.

### The pleiotropic module was highly enriched with known diagnostic markers and therapeutic targets

Having identified a link with disease development, we next assessed if the pleiotropic module was enriched for known diagnostic markers (n = 1,177) and/or therapeutic targets (n = 404) compiled from all diseases [53]. The results from these analyses revealed a significant enrichment in both markers and targets (n = 88, FE = 5.5, *P* < 10^-46^; n = 36, FE = 12.8, *P* < 10^-28^, respectively, using FET; Figure 1A). These enrichments were not confounded by the high connectivity of the pleiotropic module or by the number of genes/proteins and diseases examined (Figure S7 in Additional file 3; Table S11 in Additional file 1). Since the interaction partners of known drug targets are likely to be functionally related, we hypothesized that those partners may be novel therapeutic candidates across diseases. Indeed, examination of predicted druggable PPIs [54] in the pleiotropic module identified 173 novel therapeutic candidates (Table S18 in Additional file 1).

Since the pleiotropic module was associated with generally increased disease susceptibility, the identification of approved drugs that may significantly alter its activity could be clinically relevant. To address this hypothesis, data from the determination of the half maximal inhibitory concentration (IC50) of more than 100 drugs across 100 cancer cell lines [37] was analyzed. Although some correlations may be specific to cancer status, this dataset represents a large collection of different tissue types. Next, relative to 1,000 random sets of 158 genes compiled from the same expression dataset, several drugs were found to be frequently correlated with genes from the pleiotropic module (Figure S8 in Additional file 3; Table S19 in Additional file 1). This analysis showed 37 of the drugs significantly changed the expression of the genes in the pleiotropic module more than other genes at a FDR of 5%. Thus, those drugs may be therapeutic candidates in multiple diseases. The top significant drugs were two allosteric AKT inhibitors (AKT inhibitor VIII and MK-2206), which suggests that signaling though this kinase would be critical in different pathologies. Importantly, the pleiotropic influence of AKT function has been previously discussed in different scenarios, which include immune-related processes and pathologies.

### Disease stratification based on pleiotropic or disease-specific genes

Because the pleiotropic module was enriched for known therapeutic targets, we hypothesized that changes in the expression of pleiotropic or disease-specific genes could be used to stratify patients for response to drugs targeting these two gene categories. Briefly, we carried out prospective clinical studies of 50 patients with MS and 48 patients with SAR to define HRs and LRs to treatment. In order to identify genes that responded to treatment, CD4^+^ T cells from the patients were analyzed with gene expression microarrays before and after *in vitro* exposure of the drug. Next, we examined if genes that were shared or specific for the two diseases could separate HRs and LRs. We found that HRs and LRs could be separated by shared genes in SAR, and by MS module genes in MS.

**Figure 3:**
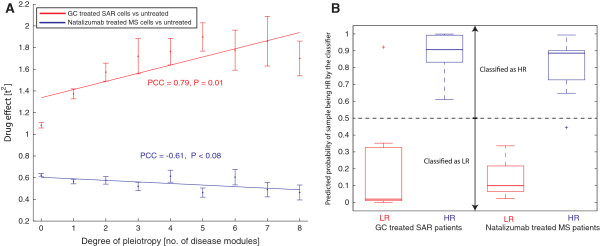
**Classification of treatment response based on pleiotropic or disease-specific genes. (A)** Glucocorticoid (GC) treatment of CD4^+^ T cells from patients with seasonal allergic rhinitis (SAR) had the largest effect on the expression of genes that participated in many disease modules. This effect was not observed following natazulimab treatment of CD4^+^ T cells from patients with multiple sclerosis (MS). The figure shows the correlation between the mean treatment ± standard error of the mean effect on mRNA expression measured by the squared student *t*-values between GC-treated and untreated cells and the number of disease modules a gene participated in. PCC, Pearson's correlation coefficient. **(B)** Pleiotropic or disease-specific genes accurately classified high and low responders to treatment in SAR and MS. The estimated probabilities (cross-validated) of a sample being a high responder (HR) based on the LASSO classifiers after drug treatment. The horizontal black line at 0.5 represent the classification border of HR and low responders (LR). The probability estimates of each group of patients are summarized into box-plots showing the median, inner quartile range, whiskers and outliers.

The 48 SAR patients were treated with GCs for two weeks during the pollen season. GCs generally reverse the expression levels of genes involved in the inflammatory response [41] and are used in the treatment of several immune diseases. The 50 MS patients were treated with natalizumab, and followed clinically during three years. Natalizumab is a drug that is mainly used in MS and specifically targets a membrane protein responsible for lymphocyte passage through the blood-brain barrier, and also influences gene expression in lymphocytes [55,56]. In both diseases, clinical specialists classified subsets of patients that were HRs and LRs (Materials and methods). The CD4^+^ T cells were obtained from untreated patients during symptom-free periods. Gene expression microarray analyses of the SAR patients showed increased likelihood of GC response for genes within many disease modules (PCC = 0.79, *P* = 0.0011; Figure 3A). By contrast, in MS, the opposite non-significant trend was found for natalizumab (PCC = -0.61, *P* = 0.079; Figure 3A).

Next, we analyzed if the disease module knowledge could be translated to prediction of treatment response for the two drugs. Therefore, we first selected only genes differentially expressed *in vitro* by the drug for further analysis (*P* < 0.05). Since genes in multiple disease modules more frequently responded to GCs, we further limited the GC classifier into genes within at least two disease modules (n = 311). Then, using the LASSO and cross-validation for model parameter fits, most patients in both diseases were correctly classified as LRs or HRs (permutation test *P* < 3 × 10^-4^; Figure 3B). Analysis of the classifiers showed that they exploited different features. The GC classifier used GC pathway (n = 29, *P* < 10^-13^, FET; see Table S20 in Additional file 1 for all pathways). By contrast, since natalizumab did not affect genes in multiple modules more frequently, we limited the MS classifier to genes in the MS module (n = 28). Conversely, the natalizumab classifier utilized genes dispersed in different pathways, and instead the main difference between HRs and LRs was due to a higher perturbation of the LRs than HRs by the drug (<t^2^_LR_ > = 1.20, <t^2^_HR_ > = 0.66, permutation test *P* < 10^-300^; Figure S9 in Additional file 3; see Table S21 in Additional file 1 for enriched pathways in the differentially expressed genes for LRs).

Thus, pleiotropic or disease-specific genes have the potential to stratify patients for drug response depending on which genes are targeted by the drug.

## Conclusions

The main findings of this study are that pleiotropic genes in T-cell-associated diseases formed a module of highly interconnected genes. This pleiotropic module generally increases disease risk and is an important source for diagnostic markers and therapeutic targets. To our knowledge, this is the first study to systematically address the functional and clinical implications of pleiotropic genes in complex diseases. Our study may have important implications for the general understanding of complex disease mechanisms and translational genomics.

The background to our study was that translational research mainly focuses on investigating disease-specific genes/proteins. Nonetheless, recent observations from GWAS have suggested an important etiological role for pleiotropic genes/proteins [4,6-8,39]. However, an important limitation in the studies of pleiotropic genes based on GWAS is that epigenetic causes may play important roles for complex disease susceptibility [9].

In this study, we reasoned that mRNA expression would be an optimal level to identify pleiotropic genes because it can be affected by both genetic and epigenetic variants. To address the problem that gene expression varies in different diseases and cell types, we searched for a common cell with pleiotropic genes based on pathway analysis of GWAS. This novel approach resulted in the identification of the Th differentiation pathway, which is expressed in CD4^+^ T cells. The Th differentiation pathway has a key role in orchestrating immune responses in autoimmune and allergic diseases [45], atherosclerosis [46], cancer [47], and obesity [48]. Our finding is in line with previous GWAS showing the importance of immune-related genes in complex diseases [5]. On the other hand, the identification of the Th pathway could be confounded by the selection of diseases analyzed with GWAS. Some definitions of pleiotropy require that the studied diseases or disease traits are seemingly unrelated, while others argue that such definitions are too subjective [57]. In this study, we addressed this potential confounder by complementary analyses. For example, to test if the identification of the Th pathway was due to the selection of diseases examined by GWAS, we repeated the analyses on somatic mutations in cancer and found that the Th pathway was significantly enriched. Next, we analyzed expression profiling data from CD4^+^ T cells from eight inflammatory and malignant diseases. In each of the diseases we could define modules of differentially expressed genes. Despite the diversity of the diseases, the modules overlapped. The overlapping part was highly interconnected and formed a pleiotropic module, which was enriched not only for the Th differentiation pathway, but also for several other inflammatory, metabolic and proliferative pathways. These pathways contained individual genes of known or recently recognized importance for multiple diseases. For example, *GATA3* is known to have a key role in Th2 differentiation in allergy, but is also a potential diagnostic marker in epithelial cancers [58]. Conversely, the tumor suppressor gene *BRCA1*, which has an important role in breast cancer, was also differentially expressed in allergen-challenged T cells. This finding is in line with *BRCA1* potentially having a role in regulating inflammation [59].

We speculated that because of the pleiotropy and interconnectivity of the pathways, the pleiotropic module would generally increase disease susceptibility. Remarkably, despite the pleiotropic module being derived from eight inflammatory and hematological diseases, it was significantly enriched with GWAS genes from all published analyses of diseases and disease traits. Thus, rather than being dispersed in the interactome, a limited number of highly interconnected genes that regulated key pathways generally increased disease susceptibility.

A confounding factor when using collections of experimental studies might be potential knowledge-related biases, that is, the experimental studies are not equally distributed among the genes. Therefore, to limit this problem we confirmed that the identified pleiotropic module genes were highly associated using databases with several other inclusion criteria, including systematic and high confidence databases. It is also important to note that the genes in the pleiotropic module were more enriched for both GWAS genes and disease phenotypes than the disease-specific genes. The findings for the GWAS genes were also replicated for cancer genes. This indicates that pleiotropic genes may have larger impact on disease phenotypes than specific genes, which has important diagnostic and therapeutic implications that are discussed below.

We then tested the potential of therapy of the pleiotropic module, and found that it was highly enriched for known diagnostic markers and therapeutic targets. Interestingly, the module also contained a large number of druggable genes that were not known drug targets. Those genes represent new therapeutic candidates across diseases. Thus, while disease-specificity is often desired for both types of molecules, our observations point to pleiotropic genes/proteins being relevant for '4P medicine' (predictive, preventative, personalized and participatory medicine [60]). Knowledge from the pleiotropic module also provides insight into disease stratification for individualized medication. Prospective clinical studies in MS and SAR combined with gene expression profiling revealed differences relative to the modular impact of the respective disease treatments. While the GCs (used in SAR) affected multiple disease-associated modules, natalizumab (used in MS) was linked to a single module. Subsequently, the expression of the pleiotropic genes was found to classify GC responders with high accuracy, while the disease-specific genes classified natalizumab responders. This observation leads us to propose that the definition of pleiotropic versus specific genes/proteins may help to identify markers for stratification, based on network molecular consequences of the therapies. Prospective studies in other diseases are required to assess this hypothesis.

Another important future research direction is to examine how the same pleiotropic genes can be associated with different diseases. Possible explanations include altered interactions between those genes, as well as between disease-specific genes. Furthermore, analyses of pleiotropic genes in other pleiotropic cells are warranted. Our pathway analysis of GWAS data indicates that there may be several different pleiotropic cells, for example, B lymphocytes, macrophages and epithelial cells. Since these cell types interact, it would be very interesting to simultaneously profile their gene expression profiles. This could help to understand disease-associated interactions on a multi-cellular scale.

In summary, given the results of this study, we propose that the pleiotropic genes/proteins warrant extensive functional and clinical studies across diverse diseases.

## Abbreviations

FE: fold enrichment; FET: Fisher's exact test; GC: glucocorticoid; GO: Gene Ontology; GWAS: genome-wide association study; HR: high responder; IPA: Ingenuity Pathway Analyses; KEGG: Kyoto Encyclopedia of Genes and Genomes; LR: low responder; MS: multiple sclerosis; PCC: Pearson’s correlation coefficient; PPI: protein-protein interaction; SAR: seasonal allergic rhinitis; SNP: single-nucleotide polymorphism; Th: T helper.

## Competing interests

The authors declare that they have no competing interests.

## Authors' contributions

MG and MB conceived the study. ME, HW, and HZ performed wet lab experiments. MG, DG, FB, CEN, JS, NB, and MAP performed the data analysis. CEN, MAP, JE, MB, JT, IK, and TO was involved in preparing figures and writing the manuscript. All authors read and approved the final manuscript.

## Supplementary Material

Additional file 1: Table S1 Manual classification of all traits and diseases in the GWAS catalogue. **Table S2.** Gene symbols of all mapped genes that harbor at least one disease-associated SNP corresponding to a disease trait. **Table S3.** All pathways for all the GWAS genes reported by IPA. **Table S4.** All KEGG pathways for all the GWAS genes. **Table S5.** Ingenuity pathways for the GWAS genes with any non-immune trait. **Table S6.** Gene symbols of all genes harboring at least one disease-associated SNP in the corresponding linkage disequilibrium block. **Table S7.** Ingenuity pathways for the genes in Table S6. **Table S8.** Gene symbols of the mapped genes with a somatic cancer mutation in the COSMIC database. **Table S9.** Ingenuity pathways for the genes in Table S8. **Table S10.** Gene symbols of the mapped genes within the OMIM database. **Table S11.** Ingenuity pathways for the genes in Table S10. **Table S12.** Statistics of all eight T-cell diseases. **Table S13.** Descriptions of the pleiotropic module genes. **Table S14.** Ingenuity pathways for the genes in Table S13. **Table S15.** Knockout mice phenotypes of the pleiotropic module genes. **Table S16.** Statistics of median degree preserving randomization of the enrichment of GWAS genes, biomarkers, therapeutic targets, cancer genes, immune mice knockout genes and pleiotropic module genes. **Table S17.** Nominal *P*-values for disease association in original GWAS of MS for all measured SNPs of the pleiotropic module genes. **Table S18.** Analysis of all druggable interactions between the pleiotropic module genes. **Table S19.** Drugs significantly associated with the expression of the pleiotropic module genes. **Table S20.** Ingenuity pathways for the GC-perturbed genes. **Table S21.** Ingenuity pathways for the genes differentially expressed in low responders of natalizumab treatment. **Table S22.** Identified disease modules for each of the studied diseases. Click here for file

Additional file 2 R code for the derivation of the disease modules, which integrate binding and expression data. Click here for file

Additional file 3: Figure S1 Pie chart of the distribution of manual classification (Table S2) of disease traits from all GWAS. **Figure S2.** The Th differentiation pathway (Ingenuity® Systems [34]), where GWAS genes (grey), growth factors (squares), transcription factors (broad ovals), complexes (standing ovals), and receptors (double standing ovals) are marked. **Figure S3.** Comparison of the overlap of genes annotated to T helper (Th) differentiation according to different data sources. **Figure S4.** The estimated probabilities (cross-validated) of a sample being a patient based on the LASSO classifiers of each of the eight T-cell diseases. **Figure S5.** The genes in the pleiotropic module had relative mean shortest paths among each other. **Figure S6.** Enrichment of nominally associated SNPs of MS for the genes in the pleiotropic module (black bars) using different cutoffs for the *P*-value for determining disease association. **Figure S7.** Mean (dots) ± standard error of the mean (bars) of the fraction of genes that are biomarkers (red) and therapeutic targets (blue) versus number of disease modules. **Figure S8.** Correlation analysis between the expression of the pleiotropic module genes and the responses of 131 drugs across cancer cell lines measured using IC50 (see Materials and methods). **Figure S9.** Differential expression of genes between MS patients before versus after *in vitro* natalizumab treatment, measured by the squared student *t*-values. Click here for file
