# Supplementary material for: Integrated genomic and prospective clinical studies show the importance of modular pleiotropy for disease susceptibility, diagnosis and treatment
Source: Genome Med. 2014 Feb 26;6(2):17. doi: 10.1186/gm534 (PMC4064311; doi:10.1186/gm534)
Supplement: Additional file 3: Figure S1 — Pie chart of the distribution of manual classification (Table S2) of disease traits from all GWAS. Figure S2. The Th differentiation pathway (Ingenuity® Systems [34]), where GWAS genes (grey), growth factors (squares), transcription factors (broad ovals), complexes (standing ovals), and receptors (double standing ovals) are marked. Figure S3. Comparison of the overlap of genes annotated to T helper (Th) differentiation according to different data sources. Figure S4. The estimated probabilities (cross-validated) of a sample being a patient based on the LASSO classifiers of each of the eight T-cell diseases. Figure S5. The genes in the pleiotropic module had relative mean shortest paths among each other. Figure S6. Enrichment of nominally associated SNPs of MS for the genes in the pleiotropic module (black bars) using different cutoffs for the P-value for determining disease association. Figure S7. Mean (dots) ± standard error of the mean (bars) of the fraction of genes that are biomarkers (red) and therapeutic targets (blue) versus number of disease modules. Figure S8. Correlation analysis between the expression of the pleiotropic module genes and the responses of 131 drugs across cancer cell lines measured using IC50 (see Materials and methods). Figure S9. Differential expression of genes between MS patients before versus after in vitro natalizumab treatment, measured by the squared student t-values. [file gm534-S3.docx]

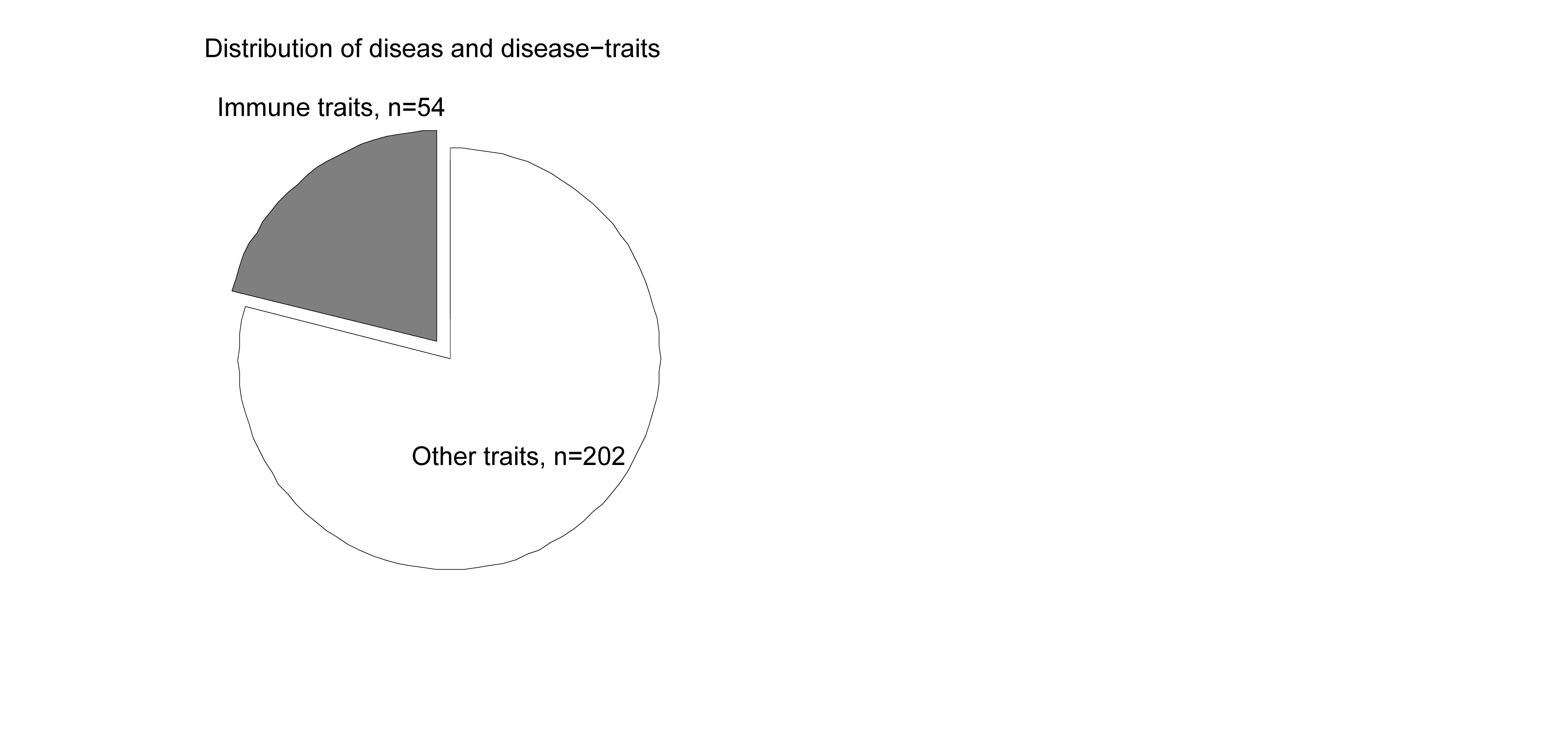


Figure S1. Pie chart of the distribution of manual classification (Table S2) of disease traits from all GWASs.


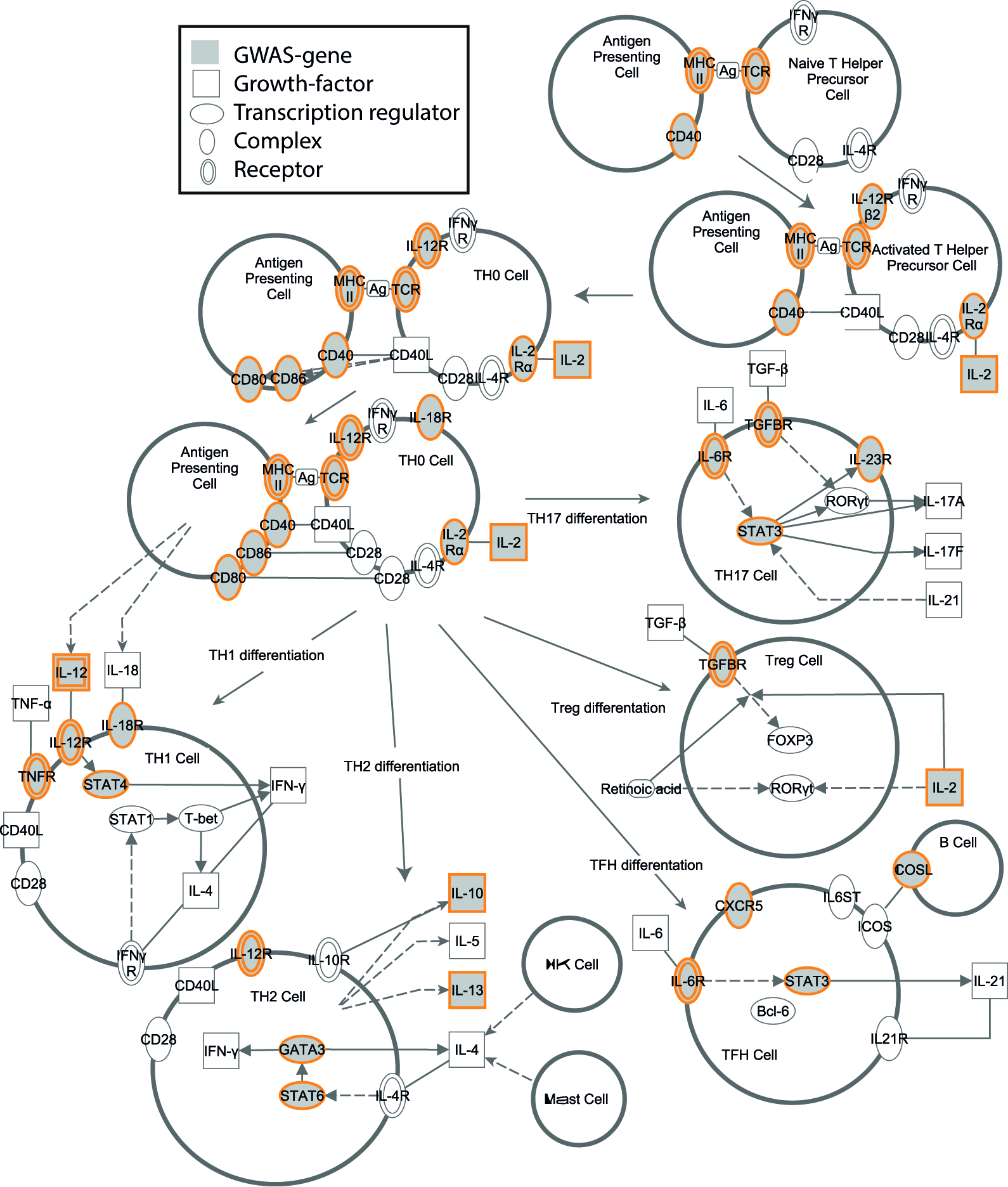


Figure S2. The Th differentiation pathway (Ingenuity® Systems, [www.ingenuity.com](http://www.ingenuity.com/)), where GWAS-genes (grey), growth-factors (squares), transcription factors (broad ovals), complexes (standing ovals), and receptors (double standing ovals) are marked.


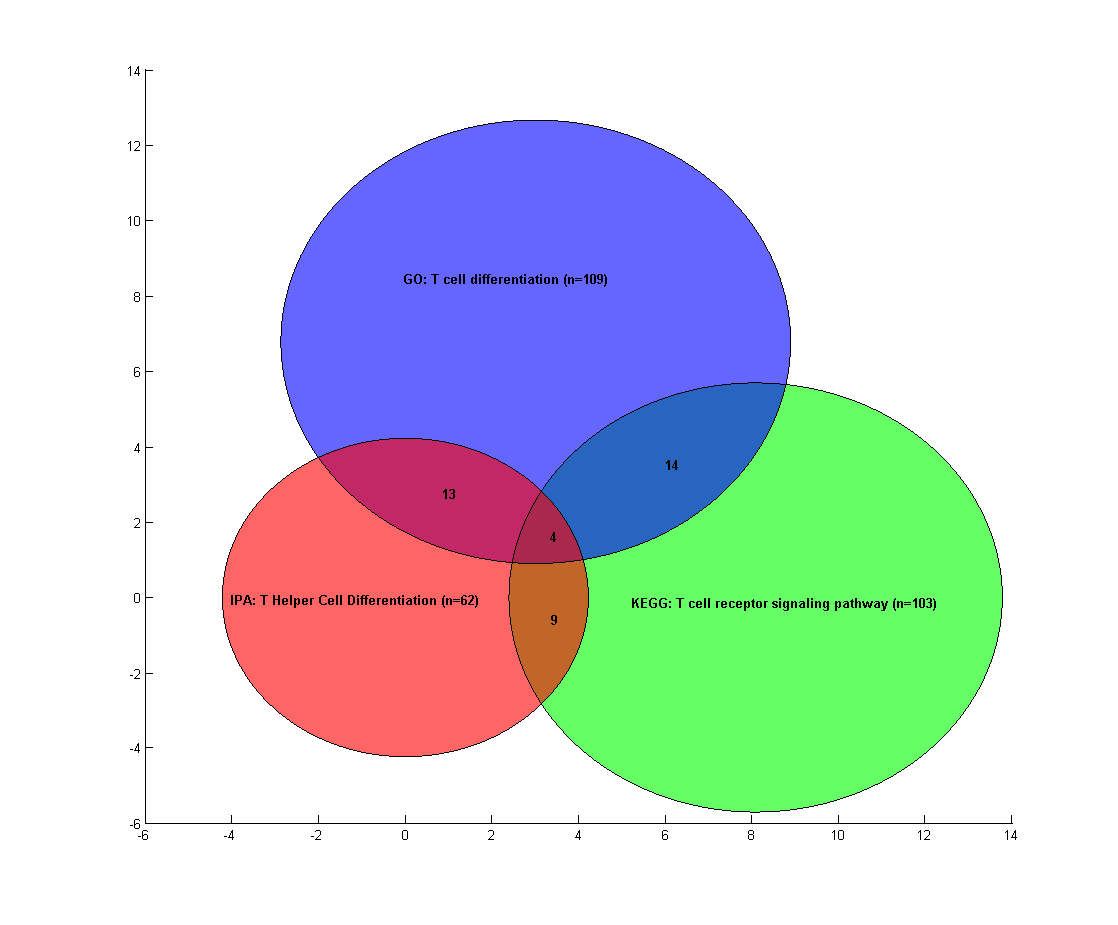


Figure S3. Comparison of the overlap of genes annotated to T helper (Th) differentiation pathway according to Ingenuity (IPA, n=62), Kyoto Encyklopedia of Genes and Genomes (KEGG, n=103), and Gene Ontology (GO, n=109). The Venn diagrams shows the total overlapping genes in the corresponding pathways. For example 4 genes were annotated to Th differentiation according to all three databases and 76 (=103-14-4-9) only in KEGG.


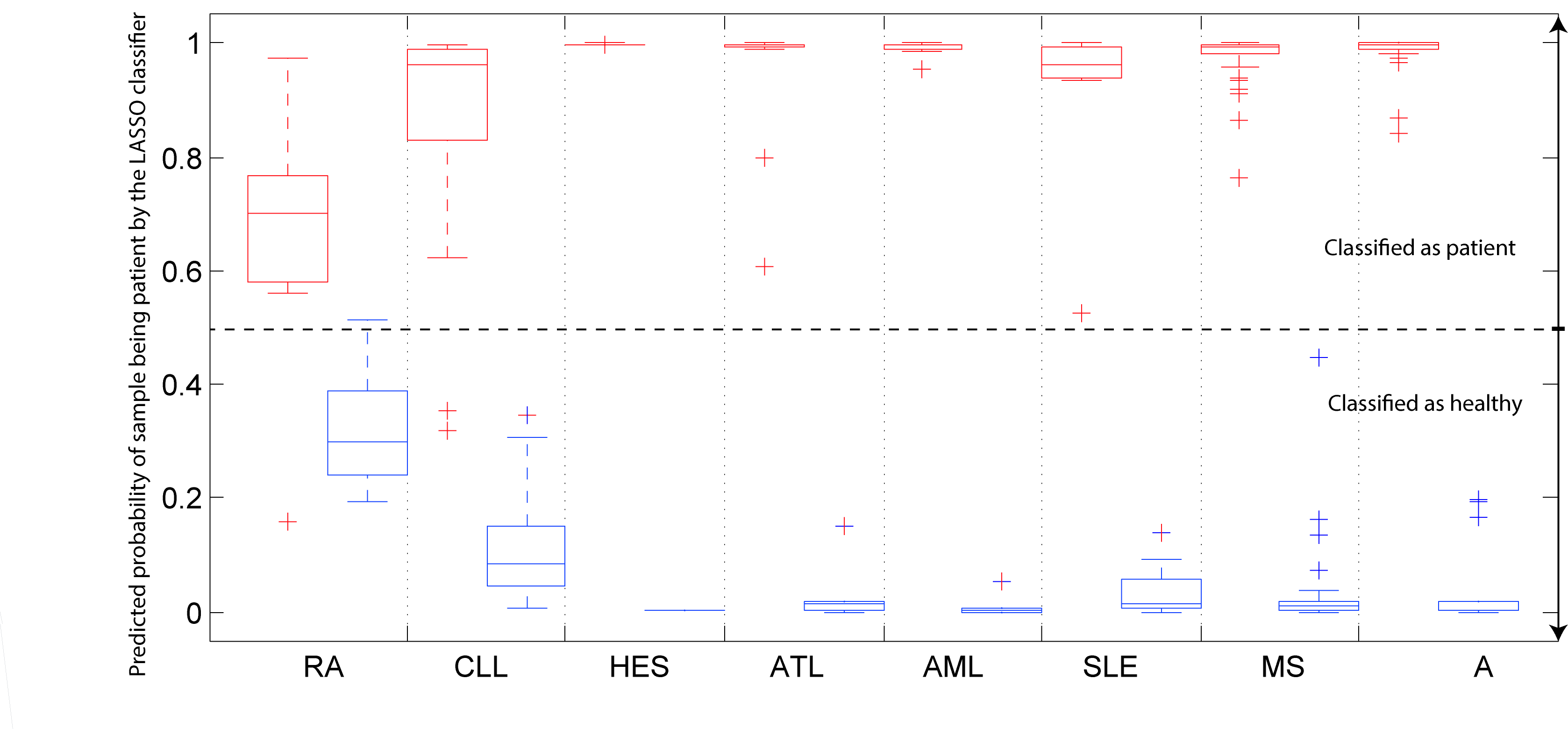


Figure S4. The estimated probabilities (cross-validated) of a sample being a patient based on the LASSO classifiers of each of the 8 T cell diseases. The horizontal black line at 0.5 are the classification boarder separating the classifications into high responders (HR) and low responders (LR). The probability estimates of each group of patients and controls were summarized into box-plots showing the median (horizontal line), inner quartile range (boxes), whiskers (dashed line) and outliers (+).


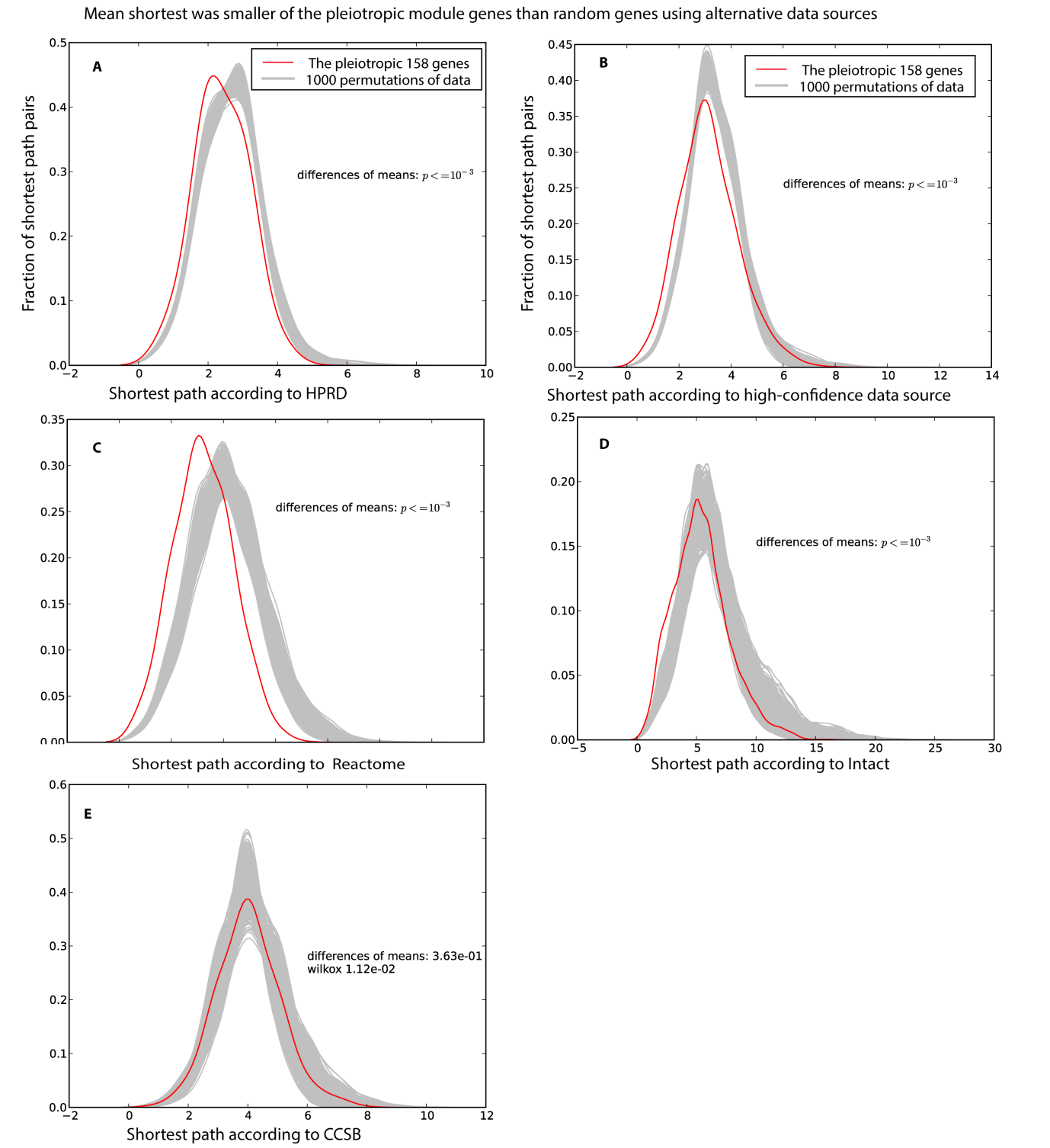


Figure S5. The genes in the pleiotropic module had relative mean shortest paths among each other. Distribution of shortest paths among the genes in the pleiotropic module versus a similar number of random genes as in the pleiotropic module. The studied databases were: HPRD [1] (A), a high confidence database [2] (B), Reactome [3] (C), Intact[4] (D), CCSB [4] (E). The P-values were calculated using a Wilcoxon rank test of 1000 permutations using the mean shortest path as test statistic. For figure A-D we had 102-136 of the 158 genes in the pleiotropic module, but only 43 were present for figure E.


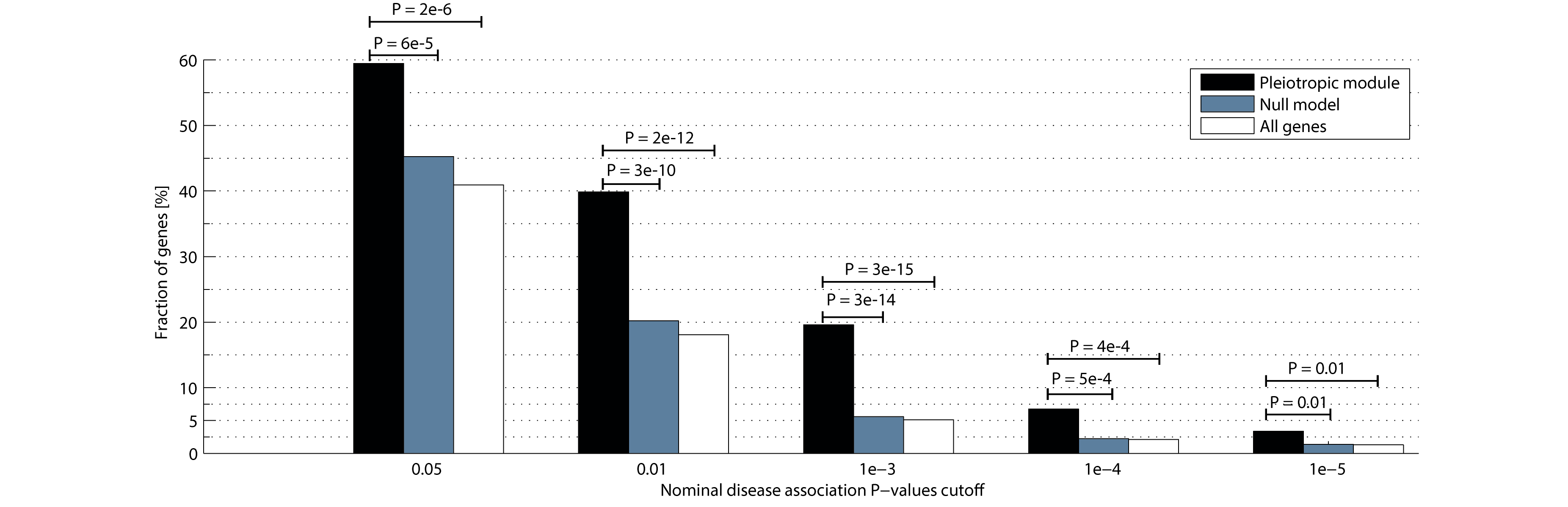


Figure S6. Enrichment of nominally associated SNPs of MS for the genes in the pleiotropic module (black bars) using different cut-offs for the P-value for determining disease association. The grey bars represent all genes with any assayed SNP in MS data, and the white bars represent the background of all genes.


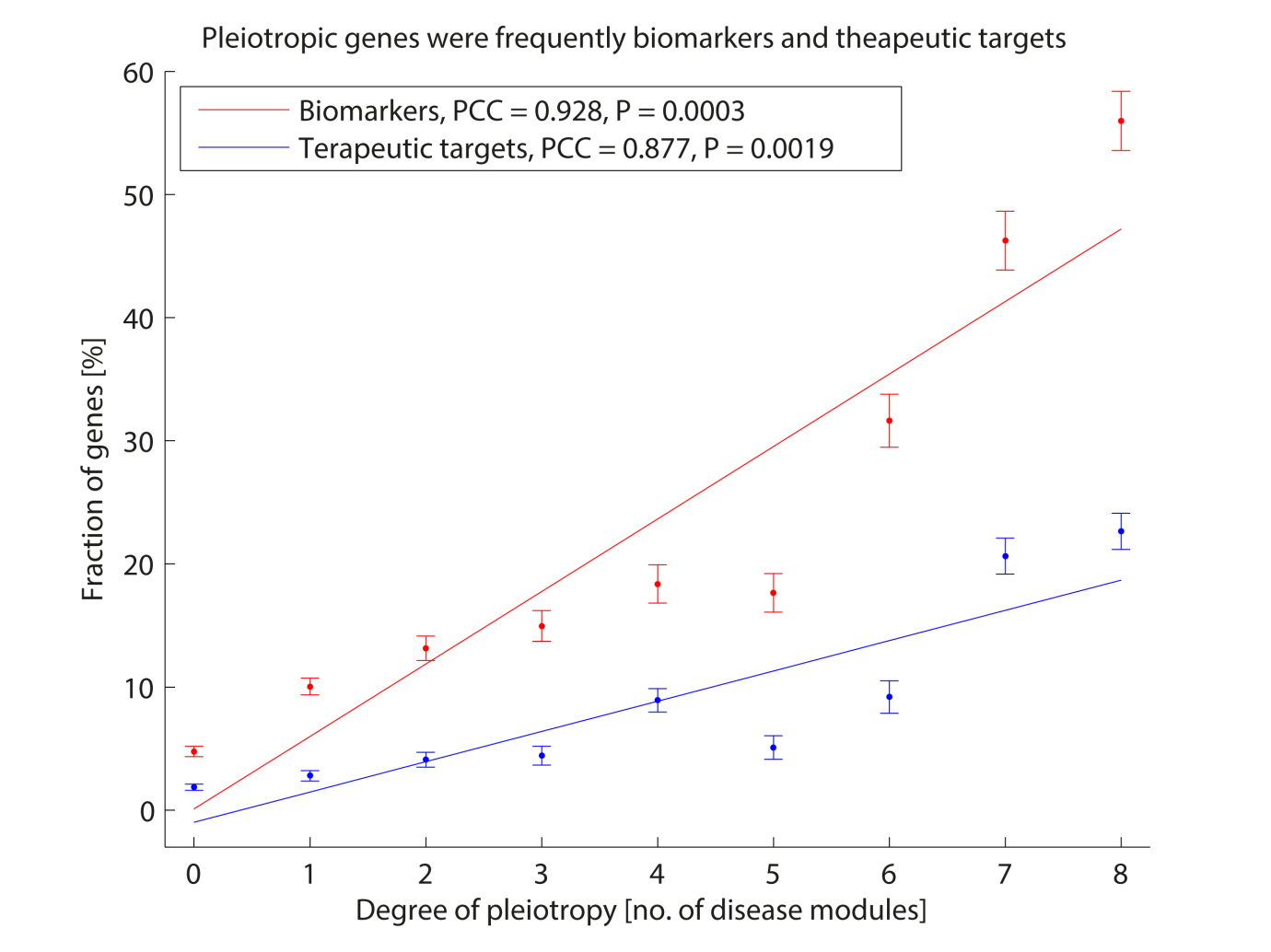


Figure S7. Mean (dots) ± SEM (bars) of the fraction of genes and that are biomarkers (red) and therapeutic targets (blue) versus number of disease modules. The correlations are high and significant for both characteristics. PCC = Pearson Correlation Coefficient.


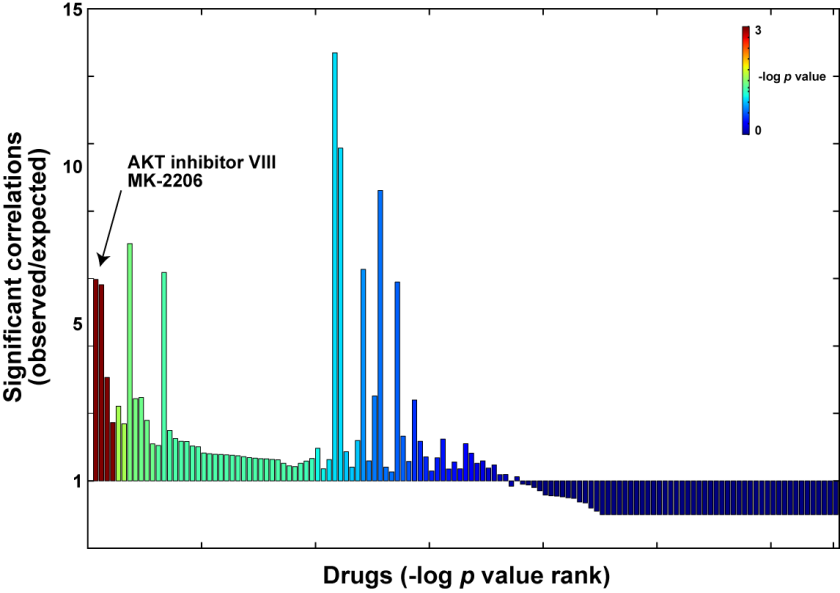


Figure S8. Correlation analysis between the expression of the pleiotropic module genes and the responses of 131 drugs across cancer cell lines measured using IC50 (see Methods).


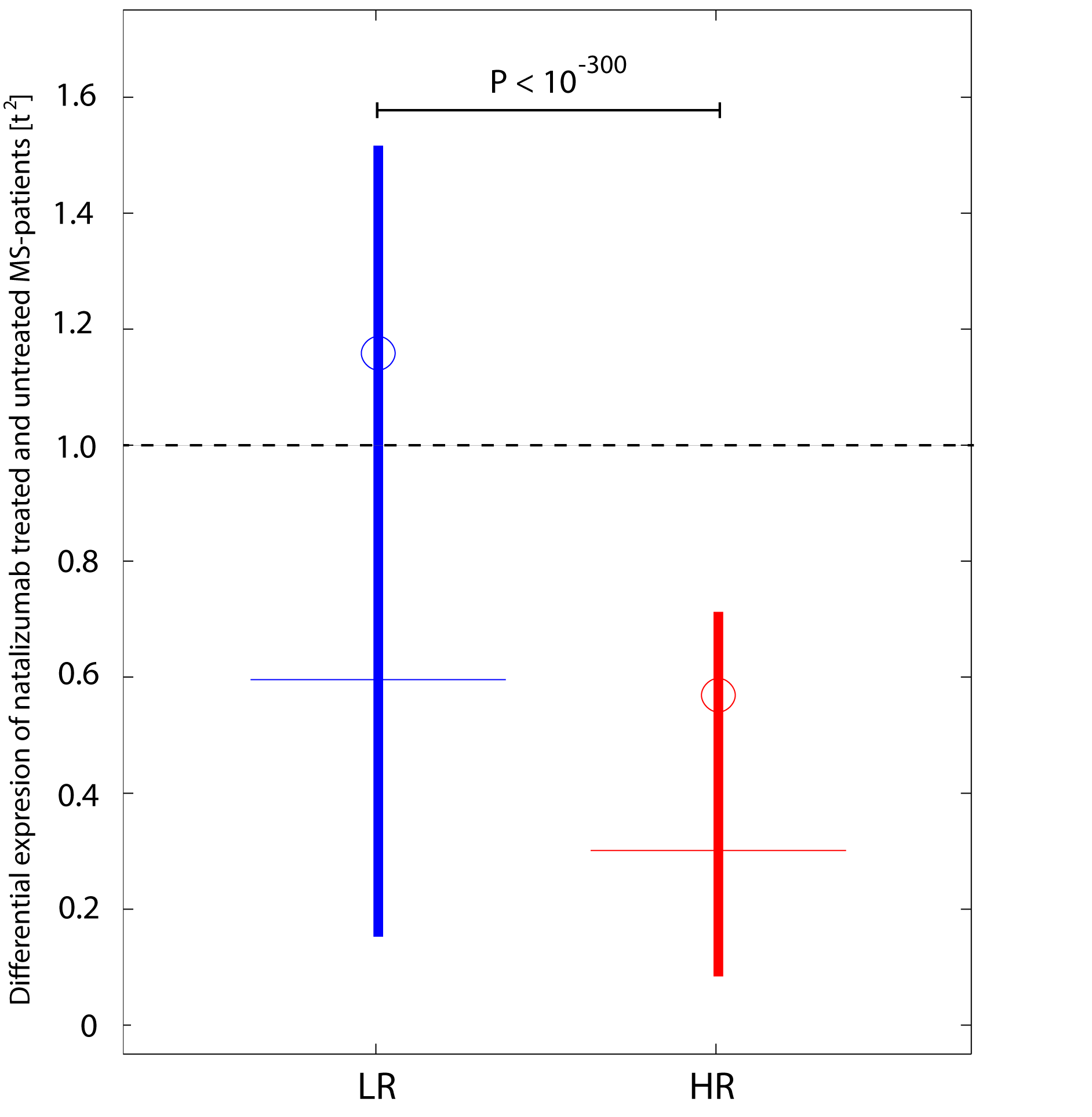


Figure S9. Differential expression of genes between MS patients before versus after *in vitro* natalizumab treatment, measured by the squared student t-values. Data are summarized for all genes in low responders (LR) and high responders (HR) to the *in vivo* treatment. The symbols depict inner quartile range (boxes), median (fat solid line), and mean (circle) of squared student t-values respectively. The black line at t^2^=1 shows the expected mean value if no difference between patients and controls were present for the gene sets. The P-value is calculated using the mean squared t –values as a test-statistic and permuting the t-values between the two groups.
